# Suicidal Thoughts, Depression, Post-Traumatic Stress, and Harmful Alcohol Use Associated with Intimate Partner Violence and Rape Exposures among Female Students in South Africa

**DOI:** 10.3390/ijerph19137913

**Published:** 2022-06-28

**Authors:** Mercilene Tanyaradzwa Machisa, Esnat Chirwa, Pinky Mahlangu, Ncediswa Nunze, Yandisa Sikweyiya, Elizabeth Dartnall, Managa Pillay, Rachel Jewkes

**Affiliations:** 1 South African Medical Research Council Gender and Health Research Unit, 1 Soutpansberg Road, Pretoria Private Bag x385, Pretoria 0001, South Africa; esnat.chirwa@mrc.ac.za (E.C.); pinky.mahlangu@mrc.ac.za (P.M.); ncediswa.nunze@mrc.ac.za (N.N.); yandisa.sikweyiya@mrc.ac.za (Y.S.); rachel.jewkes@mrc.ac.za (R.J.); 2 School of Public Health, Faculty of Health Sciences, University of Witwatersrand, 60 York Road, Parktown, Johannesburg 2193, South Africa; 3 Sexual Violence Research Initiative, 28 High Street, Waterkloof, Pretoria 0145, South Africa; elizabeth@svri.org; 4 Department of Basic Education, 222 Struben Street, Pretoria Central, Pretoria 0001, South Africa; pillay.m1@dbe.gov.za

**Keywords:** students, depression, PTSD, harmful alcohol use, suicidal thoughts, intimate partner violence, rape, childhood trauma, South Africa, mental health

## Abstract

While ample evidence from high-income country settings indicates the prevalence and risk factors for multiple mental ill-health symptoms in student populations, evidence from low- and middle-income higher education settings remains limited. We determined the frequency, associations, and structural pathways between mental health outcomes and possible risk factors among a sample of 1292 predominantly Black African and female students ages 18–30 years, enrolled at nine purposefully selected public universities and Technical Vocational Education and Training (TVET) campuses. We measured and created a mental ill-health latent outcome consisting of depressive symptoms, post-traumatic stress disorder (PTSD), and suicidal thoughts. We also measured traumatic exposures including childhood trauma, recent intimate partner violence (IPV), non-partner rape, and other life traumatic events. We used structural equation modelling to analyse data. We found that 50% of the surveyed students binge drank, 43% reported depressive symptoms, 9% reported PTSD symptoms, and 21% had suicidal thoughts. Students’ experiences of childhood trauma, food insecurity, other traumatic events, non-partner rape, and IPV impacted the mental ill-health latent. IPV experiences mediated the relationships between experiences of childhood trauma or other trauma and the mental ill-health latent, and the relationship between binge drinking and other life traumatic events. Non-partner rape mediated the relationship between food insecurity and the mental ill-health latent. Binge drinking directly impacted non-partner rape experience. The findings substantiate the need for campus-based mental health promotion, psychosocial services and treatments, and implementation of combined interventions that address the intersections of violence against women and mental health among students in South Africa.

## 1. Introduction

Mental disorders have far-reaching public health impacts, including physical health, quality of life, mortality rates, violence at the different levels of the ecological model, and other socio-economic impacts [1,2]. Women living in low- and middle-income countries, including South Africa, are disproportionately vulnerable to undetected and neglected mental ill-health [1,2,3]. Notably, South Africa has higher prevalence rates of common mental disorders when compared with other countries [4,5,6]. The largest population-based mental health survey to date estimated that one in six South Africans had mental disorders, with the most commonly occurring disorders being depression, anxiety disorders, post-traumatic stress, and substance use disorders [7].

Many studies have investigated the prevalence, risk factors, and effects of mental ill-health among student populations in high income, non-African countries, but fewer studies have been conducted in low–middle-income countries such as South Africa [1,2,8,9,10]. The global evidence shows that the prevalence of mental distress is higher in student populations compared to the general population and that female students are particularly more vulnerable compared to male students [10,11,12]. Post-traumatic stress disorder (PTSD), anxiety, depressive symptoms, substance disorders, and suicidal thoughts were highly prevalent and often comorbid, which is consistent with studies conducted with general population samples [8,10,11,12,13,14,15]. The evidence also shows that transitioning to adulthood and higher education is stressful and negatively impacts mental health [10,11,16]. Other stressors and risk factors that are identified as exacerbating students’ mental ill-health include financial problems, health-related issues, intimate relationship problems, family problems, and academic challenges [17,18]. Additionally, students’ social circumstances and trauma exposures, such as witnessing or experiencing interpersonal violence or violent crime, experiences of childhood trauma, rape, intimate partner violence, or physical assault leading to grievous bodily harm, increase the risk for depressive symptoms, PTSD, and suicidality [15,17,19,20]. The evidence also indicates that depression, PTSD, anxiety disorders, and substance use disorders negatively affects female students’ overall wellbeing, social functioning, sexual and reproductive health, academic performance, and completion of studies [8,12,14,15,16].

As mentioned previously, fewer epidemiological studies have focused on understanding the prevalence and the risk factors for comorbid mental ill-health symptoms among female students in South Africa. The most recently published South African surveys were conducted among first-year students studying at two large, urban, well-resourced, and previously White university campuses [21,22]. The surveys were part of the multi-country WHO World Mental Health International College Student (WMH-ICS) initiative [23]. The estimated rates of common mental disorders were comparable to those among students from other countries and to previous South African surveys that were conducted in the general population [21,22]. However, South African students reported a comparatively higher prevalence of lifetime suicidal ideation than students from other countries [24,25]. Similar to evidence from different global settings, the correlates or risk factors for common mental disorders among South African students included being female, being bullied, and having experienced childhood trauma, specifically emotional abuse or neglect [26,27]. Academic stressors, intimate relationship problems, and recent breakups were also associated with students’ depressive symptoms [26]. Depressive symptoms and frequent binge drinking were also associated with poor academic performance and failure [28].

While scholars have begun to study the prevalence and risk factors for mental ill-health among university students in South Africa, it is essential to acknowledge that universities constitute only a part of the national integrated post-school system [29]. South Africa has another tier of post-school public institutions in the form of Technical Vocational Education and Training (TVET) colleges, where such research is lacking. TVETs provide skills training at the intermediary level and are more accessible than universities. TVETs are located across the country, including in rural and less wealthy communities. They have lower tuition fees, and their entry requirements are less stringent—i.e., they have courses that accept learners who have only completed grade 9 and do not require completing grade 12 as needed for university entry [29,30]. Notably, the TVET sector in South Africa is growing; enrolment rates increased by 8% between 2010 and 2019 [30], gender parity was achieved, and the enrolment rates of female students far exceed that of male students [30]. Additionally, Black African students’ TVET college participation rate remains higher than that of other race groups [30].

Given this context, research on the prevalence and risk factors for multiple mental ill-health symptoms among Black African female students studying in the expanding and less-resourced TVET sector or the historically disadvantaged universities (HDUs) is lacking, but much needed. Critically, we have highlighted that previous research conducted among South African first-year university students in the better-resourced historically White universities had limitations in their consideration of violence exposures, particularly family-related trauma in adulthood and intimate partner violence (IPV) as risk factors for mental ill-health. Yet ample evidence from the South African context indicates that family-related trauma and socio-economic status are among the key drivers for mental ill-health in the general population [6,7,31,32]. Another limitation of previously published South African student studies has been that there was less focus given to investigate food insecurity and socio-economic status as risk factors for mental ill-health among female students.

This study thus seeks to answer three questions. First, “What is the frequency of mental ill-health outcomes, namely suicidal thoughts, depressive symptoms, post-traumatic stress symptoms, and substance use, among a volunteer sample of 1292 Black African, female students enrolled at selected South African public TVET colleges and HDUs?” Second, “What are the trauma-related and socio-demographic factors associated with mental ill-health symptoms?” Lastly, “How do the identified risk factors relate to each other?” The study’s ultimate objective is to investigate the associations and elucidate the structural pathways between mental health outcomes and possible risk factors.

## 2. Materials and Methods

### 2.1. Site Selection, Sample Size Calculation, and Participant Recruitment

The study employed a cross-sectional design and involved a convenience sampling approach. The survey was conducted in purposefully selected historically disadvantaged public universities located in rural South Africa and Technical Vocational Education and Training (TVET) colleges located across five provinces of South Africa. The research team purposefully selected nine campuses. Purposeful selection ensured that female students who had different socio-economic backgrounds and fields of study participated in the survey. Officials from the National Department of Higher Education and Training (NDHET) and institutional management committees were involved in the site selection process. None of the selected campuses were known to have notably higher levels of sexual violence compared to non-selected campuses.

The sample size calculation was based on the overall female student enrolment in the nine selected campuses. We assumed a prevalence of forced sex among young women of 15% based on previous research [33]. We assumed that 15% of TVET students and 10% of university students would have experienced sexual violence in the past year. We assumed that we would detect the difference in prevalence between TVET and university students at a 5% significance level and 80% power. The minimum sample size calculated was 565 TVET female students and 840 female university students.

Key staff and student leaders from each site assisted with advertising the study in their campuses. Survey marketing involved displaying posters around key locations on campuses, social media posts on institutional accounts, and circulating invites in student WhatsApp groups and through other institutional communication channels. The survey was advertised as having an aim to “understand young women’s life experiences.” The criteria for inclusion into the survey was female students ages 18–30, enrolled at an institution in the 6 months preceding the survey, and who were willing and able to participate in the study on selected days. We included undergraduate students in any year of study and excluded post-graduate students at university sites. The survey was conducted over three days per campus, between September 2018 and March 2019. Consenting female students self-completed the survey, and trained researchers were available to assist when needed. The final sample comprised 519 Black African, female TVET students and 774 Black African, female university students. Against a backdrop of a long history of apartheid and racial segregation, South African official race classifications for the population are still divided into five major racial categories: Black Africans, Whites, Indians, Coloureds, who have mixed White and Black descent, and “Other” for race groups who do not fit neatly into the four main race groups [34]. Black Africans constitute the majority of the national population, estimated at 81% at the time of the study [34].

The smaller final sample size was due to the unavailability of some students on the days selected by the research team for data collection. However, while the final sample size was lower than the calculated sample size, the study found a greater difference in past-year sexual violence prevalence between TVET and university students than was anticipated.

### 2.2. Ethics and Access

The South African Medical Research Council’s Human Research Ethics Committee approved the study (EC002-2/2018). Research approvals and site access were granted by NDHET and the management in selected institutions. Participants gave written consent before questionnaire completion. Participants’ anonymity was ensured through allocating random and unique study identification numbers to participants. The use of a self-administered questionnaire on electronic tablets allowed for participant privacy during completion, and data was directly synchronized onto the secure password-protected server where only the data manager and project managers could access it [35]. The study was conducted according to the World Health Organization’s Ethical and Safety Recommendations for Research on Domestic Violence against Women [36]. Research assistants were trained to anticipate and respond to participant distress. Participants received referral information regarding GBV-focused and psychosocial support services within their campuses and surrounds. Participants were reimbursed 50 Rands (equivalent USD 3.50) for their time.

### 2.3. Data Collection, Measurement, and Variables

The survey questionnaire was developed using the Research Electronic Data Capture (REDCap version 9.1.2, Creator Vanderbilt University, Nashville, TN, USA) platform and loaded onto electronic tablets [35]. Then, we conducted cognitive testing with fifteen female students who were not part of the main survey sample. Lastly, we used participant feedback to refine and finalise the questionnaire [37].

Table 1 shows how variables were measured in this study. The primary outcomes for this paper are mental ill-health symptoms, namely suicidal thoughts, depressive symptoms, PTSD symptoms, binge drinking, and drug use. We used measurement scales that were validated in previous South African studies [38,39]. We measured depressive symptoms using 20 items of the Centre for Epidemiologic Studies Depression (CES-D) Scale (Cronbach’s alpha = 0.88) [38,39,40]. We summed participants’ responses into a score, and a cut-off of 21+ indicated probable depression. We measured PTSD symptoms using 30 items of the Harvard Trauma Questionnaire (HTQ) (Cronbach’s alpha = 0.96) [41]. Again, participants’ responses were summed, and a cut-off of 60+ indicated PTSD symptoms. We used a single item to measure suicidal thoughts, i.e., “Have you thought of ending your life in the past four weeks?” Possible responses were yes or no. We also used three items of the Alcohol Use Disorders Identification Test (AUDIT) Scale to measure harmful alcohol use or binge drinking, which we defined as drinking five or more drinks on one occasion on a weekly or daily basis [42].

Traumatic exposures measured in the study included childhood trauma, IPV, non-partner rape, and other life traumatic events. We measured physical, sexual, emotional, IPV and non-partner rape (in the past year) using a modified version of the World Health Organization’s (WHO) Domestic Violence Questionnaire [44]. In addition, we measured childhood trauma using 14 items of the Childhood Trauma Questionnaire (Cronbach’s alpha = 0.83) [43]. We summed participants’ responses to items into a score that indicated both frequency and severity of childhood traumas. Finally, we measured other life traumatic events using ten items from the Life Events checklist and summed responses into a score [45]. We measured food availability as an indicator of socio-economic status. We measured food availability as an indicator of socio-economic status. Participants were asked three questions from the Household Hunger Scale (Cronbach’s alpha = 0.77), which is validated and was adapted to measure students’ food availability-related challenges in the month before the survey [46,47]. We summed the responses to the items to create a food insecurity score. An example of the food security scale item was “In the past four weeks, did you go a whole day and night without eating anything because there was not enough food?”.

### 2.4. Data Analysis

We conducted analyses in Mplus software version 8 (Creators Muthen & Muthen, Los Angeles, CA, USA) [48]. We assessed the internal consistency and reliability of scales using Cronbach’s alphas. Descriptive statistics was used to obtain frequencies, percentages, and means and standard deviations of variable. Chi-square tests and t-test were used to assess differences in proportion and means, respectively. Before conducting Structural Equation modelling (SEM), we used generalized linear models with a Gaussian link function to assess the relationship between depression score, PTSD score, and other exposure variables [49]. A generalized linear model with a logit link function was used to assess the relationship between binary outcomes of depressive symptoms, PTSD symptoms, and suicidal thoughts with all the exposure variables. Robust standard errors of the estimates in generalized models accounted for the clustering of students within college campuses.

In conducting the SEM, we hypothesised that depressive symptoms, PTSD, and suicidal thoughts were comorbid. We created the mental ill-health latent measurement model consisting of depressive symptoms (score), PTSD (score), and suicidal thoughts before assessing its convergent validity and composite reliability. The mental health latent had good convergent validity and reliability. Its Average Variance Extracted (AVEs) values and the Raykov Reliability Coefficient (RRC) were more than 0.7 (AVEs: 0.734; RRC: 0.733) [49,50,51]. We conducted principal component analysis of the mental health latent outcome and found good data fit (CFI: 0.999; TLI: 0.999; RMSEA < 0.001) [49,51].

**Figure 1:**
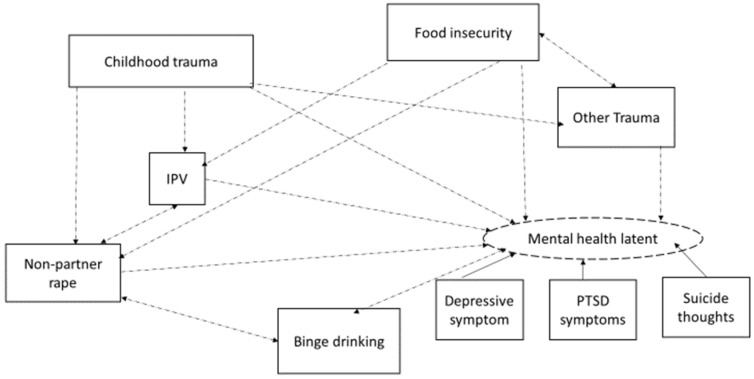
Apriori hypothesised model.

Based on existing evidence outlined in the paper introduction, our apriori hypothesis assumed that traumatic exposures, namely childhood trauma, other life traumatic events, non-partner rape, and IPV experience, were interrelated and negatively impacted the mental health latent and binge drinking (Figure 1). We also assumed that socio-economic status and food insecurity negatively impacted trauma experiences, non-partner rape, IPV experience, binge drinking, and the mental health latent outcome (Figure 1). We assumed that binge drinking would be a risky behaviour that could impact non-partner rape experience (Figure 1).

We first estimated the hypothesised an apriori path model and used WLSMV (Weighted Least Square Mean and Variance adjusted) estimators to model all available data [49]. The robust WLS estimator allows for a combination of binary, categorical, and continuous outcome variables [49]. We then used modification indices to improve the path model fit by including covariance paths where theoretically appropriate. We used the Comparative fit index (CFI); Tucker-Lewis Index (TLI), and root mean square error of approximation (RMSEA) to assess how well the SEM path model fit to observed data (CFI > 0.95; TLI > 0.95; RMSEA ≤ 0.05, indicative of good fit. The final path model indicated good fit (CFI = 0.967, TLI = 0.930, RMSEA = 0.046) [51]. The standardized coefficients with 95% confidence intervals for total, direct, and indirect effects are presented in the results section.

## 3. Results

Forty-three percent of students had depressive symptoms (CESD score ≥ 21), 8.66% had PTSD symptoms (PTSD score ≥ 60), 21% had suicidal thoughts, and 50.2% binge drank in the past year. Table 2 shows the prevalence of mental ill-health symptoms disaggregated by the different variables. We found no differences in the prevalence of mental-ill health symptoms in the two age categories. The prevalence of depressive symptoms and suicidal thoughts were higher among students from TVET colleges compared to university students. The students who experienced IPV or non-partner rape reported higher depressive symptoms, PTSD symptoms, and suicidal thoughts. Higher proportions of the students who binge drank reported more comorbid depressive symptoms, PTSD symptoms, and suicidal thoughts than those who never drank, although this was not statistically significant. The mean food security, trauma, and childhood trauma scores among students reporting depressive symptoms, PTSD symptoms, and suicidal thoughts were significantly higher than students who did not report symptoms.

Table 3 and Table 4 show the results from regression modelling to test the associations between variables and outcomes. Students who experienced IPV were twice more likely to report depressive symptoms, PTSD symptoms, and suicidal thoughts compared to those who did not experience IPV. Students who experienced non-partner rape were twice more likely to report depressive symptoms and thrice more likely to report PTSD symptoms or suicidal thoughts compared to students who did not experience non-partner rape. Higher food security, trauma, and childhood trauma scores were associated with increased risk for depressive symptoms, PTSD symptoms, and suicidal thoughts. Students who binge drank were more likely to report depressive symptoms, PTSD symptoms, and suicidal thoughts.

**Figure 2:**
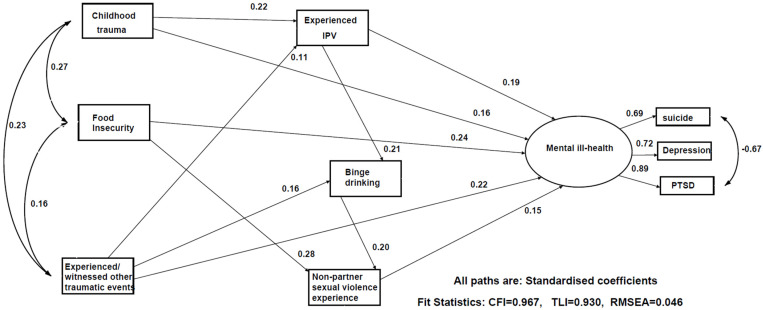
SEM final path model.

Figure 2 shows the SEM path model fit from the data. Table 5 shows the direct, indirect, and total effects between variables. Childhood trauma had direct and indirect effects on mental ill-health mediated by IPV experience. Food insecurity had direct and indirect effects on mental ill-health mediated by non-partner rape experience. Experiencing other trauma had direct and indirect effects on mental ill-health mediated by IPV experience, binge drinking, and non-partner rape. Binge drinking was associated with experiences of other trauma and IPV but had effects on non-partner rape experience. Experience of childhood trauma co-related with food insecurity and other trauma exposure. Food insecurity correlated with other trauma exposures. Experiencing IPV co-related with non-partner rape experience.

## 4. Discussion

Our study aimed to determine the frequency and factors associated with mental ill-health among predominantly Black African and younger female students (aged 18–30 years) in selected South African public TVET colleges and universities. The proportions of students reporting mental ill-health in this study indicate a high burden of mental ill-health among the surveyed female students compared to studies that were conducted among first-year students enrolled at urban, historically White universities and which used different measurement tools [21,22,25,26,27]. The goodness of fit statistics for the mental health latent outcome confirmed that depressive symptoms, post-traumatic stress symptoms, and suicidal thoughts overlap or are comorbid among some of the female students in our sample. However, binge drinking did not fit well into the latent outcome, nor did it directly correlate with the latent mental ill-health outcome in SEM. This implies that binge drinking was not necessarily comorbid with suicidal thoughts, depressive symptoms, or PTSD symptoms in this sample. These findings may be explained by evidence showing that female students often experiment with alcohol as a peer activity they partake in for enjoyment. This may be independent from the presence of mental-ill health symptoms [52]. Moreover, students’ alcohol consumption is impacted by parental modelling and socio-cultural influences that make heavy alcohol consumption acceptable in the context of entertainment, bonding, and celebrations [52]. However, the literature suggests that students exhibit more dangerous drinking patterns than non-students [53,54]. Nonetheless, our study reflects that binge drinking is risky and impacts female students ‘sexual violence experience, which directly affects mental health. This is consistent with ample evidence that has implicated alcohol abuse as being central in the complex of risky sexual behaviours among youths and students [55,56].

Our findings confirm the associations of trauma exposures as drivers of mental ill-health as found in other South African studies [32]. Female students’ experiences of childhood trauma, other traumatic events, IPV, and non-partner rape, which are in part driven by inequitable gender beliefs, directly impacted mental ill-health. Experiencing other trauma and IPV also impacted binge drinking. Most importantly, our findings show the central role of IPV in mediating or moderating the relationship between different variables and mental ill-health among female students in higher education settings. The results also confirmed that students’ food insecurity while on campus is problematic and poses direct and indirect mental ill-health risks through a path mediated by sexual violence experience, as shown elsewhere [3].

These findings show the potential impact of violence prevention programming and response in reducing and mitigating the high burden of mental ill-health among female students in South African higher education settings. Improving female students’ mental health and wellbeing will require interventions that address risk factors for IPV and rape, including experiences of childhood trauma. Group-based, gender-transformative interventions that employ participatory approaches and critical reflection, and are delivered to both men and women have been effective in shifting inequitable gender norms and reducing GBV in South African community settings [57,58]. The adaptation of effective interventions addressing GBV perpetration by male students is crucial for the South African higher education sector. Considering these findings amongst female students, it must be acknowledged that some female students’ intimate partners are young men studying in higher education who may also be facing high levels of mental ill-health. Therefore, future research to understand the prevalence, risk factors, and interventions to address mental ill-health among male students is critical.

Apart from the experiences of trauma measured in this study, life in higher education and the associated academic pressures are stressful and impact mental wellbeing amongst female students [11,28]. These impacts have since been compounded by the COVID-19 pandemic, including the changes in the teaching and learning models that needed to be adopted [59]. Our findings point to the need to prioritise and reconfigure the delivery of survivor-friendly psychosocial and student counselling services within higher education settings in South Africa. Personnel working in student counselling services must be trained to address the multiple factors associated with mental ill-health, including those we identified in this study, i.e., trauma, financial strain, food insecurity, impending COVID-19, and other academic-related stressors [59]. Other scholars have argued for innovation and online delivery of counselling services to cater to students’ needs, most critical during the COVID-19 pandemic [59]. Additionally, strengthened referral pathways to health care for the treatment of more severe psychopathology are necessary [1].

On the other hand, there is evidence that interventions promoting mental health and enhancing social and emotional coping skills implemented outside of healthcare settings can effectively address less-severe symptomatology [1]. The World Health Organisation has developed universal, lay-counsellor delivered mental health interventions that have been successfully adapted and found effective in addressing multiple mental ill-health symptoms among trauma-exposed populations in diverse country settings [60,61]. Researchers must build on effective universal, low-cost mental health interventions by culturally and contextually adapting content for delivery to students in resource-constrained South African higher education settings [21]. Moreover, implementation research focused on innovative intervention approaches and delivery modalities, including either physical or online, or blended approaches, must be prioritised to evaluate programme effectiveness in averting the negative impacts of mental ill-health on overall health, academic performance, and outcomes among students. Research is also needed to deepen understanding of what works to provide remote mental health support, and what strategies work best to build successful therapeutic alliances when services or interventions are delivered to students in the post-COVID era where face-to-face service delivery has been challenged [62].

We found that food insecurity negatively impacted mental ill-health and non-partner rape. Notably, students in the more resource-constrained TVET colleges reported a higher prevalence of depressive symptoms, PTSD symptoms, and suicidal thoughts than university students, even though the South African government currently provides NSFAS bursaries to mitigate resource and food insecurity challenges encountered by students from low-income households [25]. Other researchers found that students do not always manage these funds well [63]. The food insecurity reported by some students in this study could indicate poor financial management skills and how to budget and manage NSFAS bursary funds. Students’ lack of food may also be due to them remitting some of their bursary funds to support their impoverished families [64,65]. Therefore, mitigating the material risks faced by female students and the associated mental health outcomes will require upskilling them in basic personal finance management [63]. For some students, challenges relating to the late payment of NSFAS bursary funds contribute to young women’s engagement in risky sexual behaviours as a coping strategy while waiting to receive the funds [66]. This suggests that the government’s provision of bursaries without addressing financial management skills, broader structural inequalities, and poverty faced by the previously disadvantaged Black African population will not directly benefit students in higher education. Other studies found that the relationships between socio-economic variables and sexual violence experience were mediated by risky sexual behaviours, mainly through engagement in transactional sex or having multiple sexual partners [55]. Finally, access to mental health services for all South Africans is minimal, and access for students to quality mental health care is also extremely limited. Any work addressing mental health care and support needs to acknowledge this critical gap in primary mental health services [67,68].

This study has limitations in that we used self-reported measures of mental ill-health. However, the instruments used are validated for use in the South African context. In addition, the survey has a cross-sectional design which limits our understanding of causality and temporality. Furthermore, the convenience and purposeful sampling of TVET and HDU sites limit the generalisability of these findings to all female students in South African higher education. However, the data on risk factors for mental ill-health is valuable to inform intervention development. The study is also limited because the influences of academic and other life stressors were not measured or adjusted for in the analyses. Therefore, the associations reported in this article only pertain to and must be interpreted within the confines of measured variables.

## 5. Conclusions

Our study has highlighted the high burden of mental ill-health among volunteer Black African female students enrolled at South African public TVET colleges and HDUs, which is associated with multiple risk factors. The findings confirm the associations of trauma exposures as drivers of mental ill-health among female students. They implicate recent IPV experience as a significant risk factor, mediator, and moderator in the relationships between different variables and mental ill-health. They necessitate the implementation of innovative, low-cost, contextual, culturally relevant, campus-based, and combined interventions that address the intersections of GBV and mental health among TVET college and HDUs students in South Africa. Interventions must also address the multiple risk factors at the different levels of the social ecology.

## Figures and Tables

**Table 1:** Variable Measurement.

Variables	Observed Measures	Variable Description	Number of Items and Measurement Tool/Description	Examples of Measurement Items	Cronbach’s Alpha
**Latent outcome** Mental ill-health	Depressive symptoms	Score, 21+ cut-off, binary	Twenty items of the Centre for Epidemiologic Studies Depression (CES-D) Scale [38,39,40]	Bothered by things that usually don’t bother, unable to cheer up even with the help of family or friends. *Responses*: Rarely or none of the time, Some or a little of the time, Moderate amount of time, Most or all of the time	0.88
	PTSD symptoms	Score, 60+ cut-off, binary	Thirty items of the Harvard Trauma Questionnaire [38,41].	Experiencing recurrent thoughts or memories of most hurtful or terrifying events, feeling as though the event is happening again. *Responses* Not at all, A little, Quite A Bit, extremely	0.96
	Suicidal thoughts	binary	Single question	In the past four weeks, has the thought of ending your life been in your mind? *Responses* Yes/No	n/a
**Observed variable**	Binge drinking/Harmful alcohol use	categorical	Three items of the AUDIT C Scale to [42]	How often do you drink five or more alcoholic drinks on the same day? *Responses*: Never, Occasionally, Monthly, Weekly, Daily, or almost daily	n/a
**Observed variable**	Childhood trauma	score, binary	Fourteen items of the Childhood Trauma Questionnaire [43]	Before I turned 18, I was forced to have sex with a man when I did not want to. *Responses*: never (= 0), sometimes (= 1), often (= 2), very often (= 3).	0.83
**Observed variable** Past year physical, sexual, or emotional IPV	Past year physical IPV	binary	Five items on experiences of physical IPV using WHO Domestic Violence Questionnaire [44]	Has a boyfriend/husband ever slapped you or thrown something at you which could hurt you? Has any of this happened in the past 12 months? *Responses* Yes/No	n/a
	Past year emotional IPV	binary	Four items on experiences of emotional IPV using WHO Domestic Violence Questionnaire [44]	Has a boyfriend/husband ever insulted you or made you feel bad about yourself? How often has this happened? Has any of this happened in the past 12 months? *Responses* Yes/No	n/a
	Past year sexual IPV	binary	Three items including forced sex or sexual acts using WHO Domestic Violence Questionnaire [44]	Have you ever had sex with a boyfriend/husband when you didn’t want to because he physically forced or threatened or pressured you? Did this happen many times, more than once, a few times, once, or did it not happen?	n/a
**Observed variable**	Past year non-partner rape	binary	Four items about forced sex by a male non-partner using WHO Domestic Violence Questionnaire [44]	How many times were you forced to have sex by someone who was not your boyfriend/husband? *Responses* Never, Once, More than one time	n/a
**Observed variable**	Other life trauma	Score	Five items of the Life Events checklist [45]	Have you ever experienced any of the following: witnessed the murder of family or friend? *Responses*: Yes/No	n/a
**Observed variable**	Food security	score	Three adapted items from the Household Hunger Scale [46,47]	In the past four weeks, did you go a whole day and night without eating anything because there was not enough food? *Responses*: 0 = Not at all, 1 = Rarely, 2 = Sometimes, 3 = Often	0.77

**Table 2:** Prevalence of depression, PTSD, and suicidal thoughts disaggregated by variables.

		Depression			PTSD			Suicidal Thoughts			
	Total n = 1293	No Depression n = 728	Depression n = 565		PTSD Score <60 n = 1181	PTSD Score > = 60 n = 112		No Suicidal Thoughts n = 1002	Suicidal Thoughts n = 267	Missing n = 24	
	n	n (%)	n (%)	*p*-Value	n (%)	n (%)	*p*-Value	n (%)	n (%)	n (%)	*p*-Value
**Age group**											
18–24	1072	603 (56.3)	469 (43.7)	0.871	982 (91.6)	90 (8.4)	0.176	829 (77.3)	221(20.6)	22 (2.1)	0.723
25–30	200	112 (56)	88 (44)		178 (89)	22 (11)		155 (77.5)	43 (21.5)	2 (1)	
Missing age	21	13 (61.9)	8 (38.1)		21(100)	0 (0)		18 (85.7)	3 (14.3)	0 (0)	
**Type of institution**											
Tvet college	519	267 (51.5)	252 (48.5)	0.004	476 (91.7)	43 (8.3)	0.693	353 (68)	156 (30.1)	10 (1.9)	<0.0001
University	774	461 (59.6)	313 (40.4)		705 (91.1)	69 (8.9)		649 (83.9)	111 (14.3)	14 (1.8)	
**Past 12 m IPV experience (n = 1216)**											
No	692	444 (64.2)	248 (35.8)	<0.0001	654 (94.5)	38 (5.5)	<0.0001	577 (83.4)	105 (15.2)	10 (1.5)	<0.0001
Yes	524	246 (46.9)	278 (53.1)		459 (87.6)	65 (12.4)		375 (71.6)	141 (26.9)	8 (1.5)	
**Experienced non-partner rape**											
No	1196	690 (57.7)	506 (42.3)	<0.0001	1104 (92.3)	92 (7.7)	<0.0001	944 (78.9)	231 (19.3)	21 (1.8)	<0.0001
Yes	97	38 (39.2)	59 (60.8)		77 (79.4)	20 (20.6)		58 (59.8)	36 (37.1)	3 (3.1)	
**Alcohol use**											
None	593	361 (60.9)	232 (39.1)	0.070	561 (94.6)	32 (5.4)	0.067	484 (81.6)	106 (17.9)	3 (0.5)	0.113
yes, but no binge drinking	201	117 (58.2)	84 (41.8)		182 (90.6)	19 (9.4)		156 (77.6)	42 (20.9)	3 (1.5)	
yes, occasional binge drinking	351	185 (52.7)	166 (47.3)		318 (90.6)	33 (9.4)		269 (76.6)	79 (22.5)	3 (0.9)	
Yes, regular binge drinking	97	51 (52.6)	46 (47.4)		88 (90.7)	9 (9.3)		70 (72.2)	26 (26.8)	1 (1.03)	
Missing	51	14 (27.5)	37 (72.5)		32 (62.8)	19 (37.3)		23 (45.1)	14 (27.5)	14 (27.5)	
	**mean (sd)**	**mean (sd)**	**mean (sd)**	***p*** **-value**	**mean (sd)**	**mean (sd)**	***p*** **-value**	**mean (sd)**	**mean (sd)**		***p*** **-value**
**Food security score** n = 1276	7.51 (0.16)	6.13 (0.19)	9.32 (0.26)	<0.0001	7.19 (0.16)	11.06 (0.69)	<0.0001	6.96 (0.18)	9.55 (0.40)		<0.0001
**Other life trauma score** n = 1276	1.56 (0.05)	1.22 (0.06)	2.02 (0.08)	<0.0001	1.51 (0.05)	2.29 (0.23)	<0.0001	1.36 (0.05)	2.33 (0.13)		<0.0001
**Childhood trauma score** (n = 1274)	5.31 (0.13)	4.43 (0.14)	6.44 (0.22)	<0.0001	5.06 (0.12)	8.01 (0.61)	<0.0001	4.95 (0.13)	6.58 (0.36)		<0.0001

**Table 3:** Bivariate linear regression of mental-ill health score outcomes and variables.

	Depression Score Outcome			PTSD Score Outcome		
	Coef.	95% CI	*p*-Value	Coef.	95% CI	*p*-Value
Experience IPV in past year	4.3	3.21 to 5.40	<0.001	9.71	7.38 to 12.04	<0.001
Experience non-partner sexual violence in past year	4.42	1.77 to 7.06	0.001	13.92	9.1 to 18.74	<0.001
Food insecurity score	0.61	0.45 to 0.76	<0.001	1.00	0.75 to 1.26	<0.001
Childhood trauma score	0.58	0.45 to 0.72	<0.001	1.46	1.27 to 1.66	<0.001
Experienced other trauma (score)	1.36	1.08 to 1.64	<0.001	3.33	2.57 to 4.1	<0.001
Alcohol use:						
none	Ref			Ref		
yes, but no binge drinking	1.33	−0.10 to 2.76	0.067	2.59	0.63 to 4.54	0.009
yes, occasional binge drinking	2.06	1.07 to 3.04	<0.001	5.15	3.01 to 7.30	<0.001
Yes, regular binge drinking	1.47	−0.79 to 3.72	0.202	6.89	3.49 to 10.28	<0.001

**Table 4:** Bivariate logistic regression of mental-ill health binary outcomes and variables.

	Depression—Binary Outcome			PTSD—Binary Outcome			Suicidal Thoughts		
	OR	95% CI	*p*-Value	OR	95% CI	*p*-Value	OR	95% CI	*p*-Value
Experience IPV in past year	2.02	1.70 to 2.39	<0.001	2.4	1.79 to 3.23	<0.001	2.07	1.65 to 2.61	<0.001
Experience non-partner sexual violence in past year	2.13	1.43 to 3.18	<0.001	3.15	1.94 to 5.10	<0.001	2.53	1.81 to 3.54	<0.001
Food insecurity score	1.10	1.07 to 1.14	<0.001	1.11	1.07 to 1.14	<0.001	1.08	1.03 to 1.12	<0.001
Childhood trauma score	1.11	1.09 to 1.14	<0.001	1.11	1.07 to 1.15	<0.001	1.08	1.04 to 1.11	<0.001
Experienced other trauma (score)	1.31	1.25 to 1.37	<0.001	1.21	1.08 to 1.36	0.001	1.32	1.23 to 1.42	<0.001
Alcohol use:									
none							Ref		
yes, but no binge drinking	1.11	0.92 to 1.34	0.291	1.84	1.10 to 3.06	0.019	1.23	0.98 to 1.56	0.077
yes, occasional binge drinking	1.39	1.12 to 1.74	0.004	1.76	1.05 to 2.96	0.033	1.34	0.99 to 1.82	0.059
Yes, regular binge drinking	1.41	0.95 to 2.10	0.091	1.79	1.12 to 2.87	0.016	1.69	1.16 to 2.47	0.007

**Table 5:** SEM Output Table.

	Direct Effects		Indirect Effects		Total Effects	
	Standardised Coef (95% CI)	*p*-Value	Standardised Coef (95%CI)	*p*-Value	Standardised Coef (95% CI)	*p*-Value
Mental Health issues ← Experience/witness traumatic events	0.219 (0.170, 0.267)	<0.001	0.025 (0.009, 0.041)	0.004	0.244 (0.195, 0.292)	<0.001
Mental health issues ← Childhood trauma	0.164 (0.108, 0.220)	<0.001	0.043 (0.019, 0.066)	0.001	0.207 (0.155, 0.259)	<0.001
Mental health issues ← Food insecurity	0.238 (0.175, 0.301)	<0.001	0.042 (0.007, 0.076)	0.023	0.280 (0.228, 0.331)	<0.001
Mental Health issues ← IPV experience	0.188 (0.102, 0.273)	<0.001			0.188 (0.102, 0.273)	<0.001
Mental Health issues ← Non-partner sexual violence experience	0.149 (0.041, 0.257)	0.007			0.149 (0.041, 0.257)	0.007
Binge drinking ← Experience/witness traumatic events	0.156 (0.097, 0.215)	<0.001	0.022 (0.005, 0.039)	0.012	0.178 (0.120, 0.237)	<0.001
Binge drinking ← IPV Experience	0.210 (0.131, 0.289)	<0.001			0.210 (0.131, 0.289)	<0.001
Non-partner sexual violence experience ← Food insecurity	0.280 (0.180, 0.380)	<0.001			0.280 (0.180, 0.380)	<0.001
Non-partner sexual violence experience ← Binge drinking	0.200 (0.080, 0.320)	0.001			0.200 (0.080, 0.320)	0.001
IPV experience ← Experience/witness traumatic events	0.105 (0.033, 0.178)	0.004			0.105 (0.033, 0.178)	0.004
IPV experience ← Childhood trauma	0.220 (0.147, 0.293)	<0.001			0.220 (0.147, 0.293)	<0.001
**Covariances**	**Standardised Coef. (95%CI)**	***p*** **-value**				
Childhood trauma and food insecurity	0.271 (0.223, 0.320)	<0.001				
Childhood trauma and other life traumas	0.228 (0.180, 0.275)	<0.001				
Food insecurity and other life traumas	0.158 (0.103, 0.213)	<0.001				
IPV Experience and non-partner violence experience	0.330 (0.185, 0.474)	<0.001				
PTSD and suicidal thoughts						

Fit Statistics: CFI = 0.967; TLI = 0.930; RMSEA = 0.046.

## Data Availability

The authors have made the minimal dataset available as a Supplementary Materials to this submission.

## Supplementary Materials

The following supporting information can be downloaded at: https://www.mdpi.com/article/10.3390/ijerph19137913/s1, Table S1: Minimal Data Set.
